# Beyond Captions: Linking Figures with Abstract Sentences in Biomedical Articles

**DOI:** 10.1371/journal.pone.0039618

**Published:** 2012-07-18

**Authors:** Joseph P. Bockhorst, John M. Conroy, Shashank Agarwal, Dianne P. O’Leary, Hong Yu

**Affiliations:** 1 Department of Computer Science, University of Wisconsin–Milwaukee, Milwaukee, Wisconsin, United States of America; 2 IDA/Center for Computing Sciences, Bowie, Maryland, United States of America; 3 Department of Health Sciences, University of Wisconsin–Milwaukee, Milwaukee, Wisconsin, United States of America; 4 Computer Science Department and UMIACS, University of Maryland, College Park, Maryland, United States of America; The Centre for Research and Technology, Hellas, Greece

## Abstract

Although figures in scientific articles have high information content and concisely communicate many key research findings, they are currently under utilized by literature search and retrieval systems. Many systems ignore figures, and those that do not typically only consider caption text. This study describes and evaluates a fully automated approach for associating figures in the body of a biomedical article with sentences in its abstract. We use supervised methods to learn probabilistic language models, hidden Markov models, and conditional random fields for predicting associations between abstract sentences and figures. Three kinds of evidence are used: text in abstract sentences and figures, relative positions of sentences and figures, and the patterns of sentence/figure associations across an article. Each information source is shown to have predictive value, and models that use all kinds of evidence are more accurate than models that do not. Our most accurate method has an 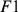-score of 69% on a cross-validation experiment, is competitive with the accuracy of human experts, has significantly better predictive accuracy than state-of-the-art methods and enables users to access figures associated with an abstract sentence with an average of 1.82 fewer mouse clicks. A user evaluation shows that human users find our system beneficial. The system is available at http://FigureItOut.askHERMES.org.

## Introduction

The rapid growth of electronic full-text biomedical articles has enabled the development of information systems that allow researchers to search and navigate large literature databases. Key content of many articles resides in images, charts, plots, tables or diagrams, and there is considerable interest in developing new figure aware systems. Because of the important role of figures, they often are referred to and discussed explicitly and implicitly throughout an article. However, nearly all existing systems for figure search rely solely on the text in captions, and thus fail to consider other key document elements. We present novel algorithms for automatically “linking” or “associating” sentences in the abstract of a scientific article with figures in the article body. These and related methods will help figures become a key part of next generation search systems. We use the terms “associating” and “linking” to indicate that a figure and a sentence in the abstract are related. In particular, the figure gives supporting information for the sentence in the abstract. This use of these terms is common in data mining and text analysis. It should not be confused with genetic, biological or medical uses of the terms “links” and “association”.

Our approach uses three types of evidence to predict whether or not an abstract sentence is associated with a figure. The first type of evidence is text. While the textual representation of a sentence is simply the terms in the sentence, the appropriate textual representation of a figure is not so clear. We investigate textual figure representations based on terms in the figure’s caption and/or its referencing paragraphs. We use probabilistic language models to assess the textual similarity between an abstract sentence and a figure. The second type of evidence is the relative positions of a sentence and figure. Previous work by our group [1] has shown that sentences at the beginning of an abstract are more likely to be associated with figures near the beginning of an article, middle sentences are more likely to be associated with middle figures, and so on. We use probabilistic distance models to reason about the relative positions for both linked and non-linked instances. The third type of evidence is patterns of sentence/figure links across an article. Since the presence or absence of a link for one instance can affect the likelihood of a link for other instances [1], we introduce novel approaches for representing linkage patterns based on hidden Markov models (HMMs) [2] and conditional random fields (CRFs) [3].

**Figure 1:**
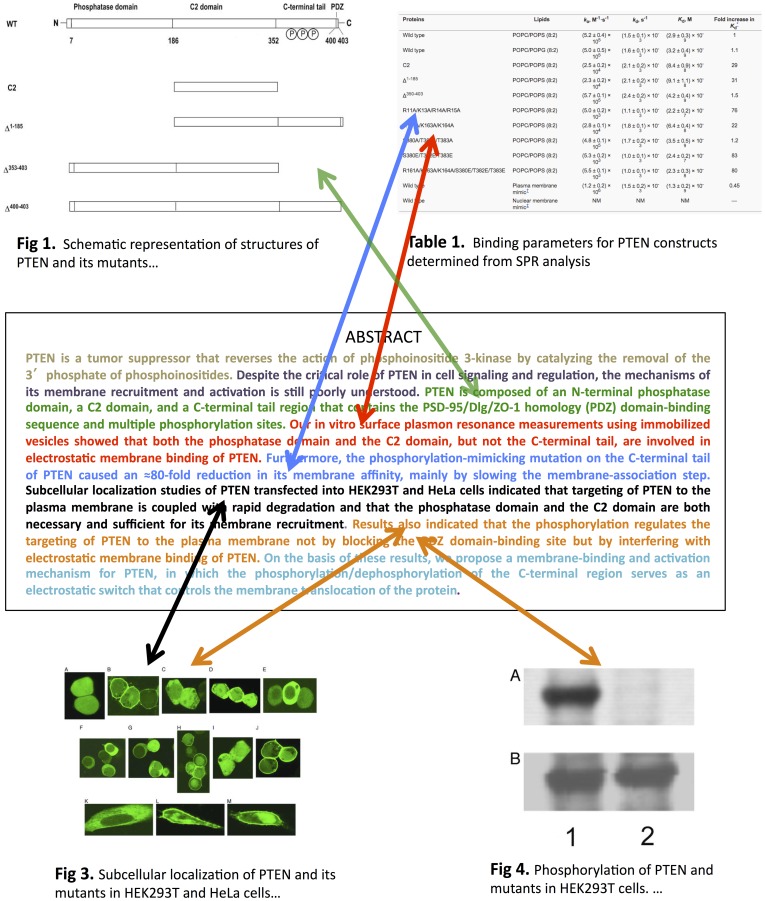
An example of a full-text biomedical article (pmid = 12808147) with author identified links between sentences in the abstract and figures and tables in the body of the article. Abstract sentences are shown in different colors. Arrows denote the annotated associations and arrow colors correspond to sentence color. To save space, figure captions are truncated and Fig. 2, which is not linked with any sentence, is not shown. (Figures republished with permission from [32], Copyright (2003) National Academy of Sciences, U.S.A.).

Our experimental evaluation uses a corpus of 114 biomedical articles annotated by their authors for all links between abstract sentences and figures. Figure 1 shows the annotated linkages between figures and the abstract sentences of one such article. We use supervised learning to learn language, distance, and linkage flow models, and use probabilistic methods to effectively combine predictions of the three models. Cross-validation experiments are used to evaluate our methods. The key findings are (i) each type of evidence has predictive value, (ii) predictions of models that combine evidence sources are more accurate than the predictions of models that use a single evidence source, (iii) across articles the average maximum F1 score of our combined approach is 69%, and (iv) our predictions would save users an average of 1.82 mouse clicks when searching for a figure associated with an abstract sentence in a conceptualized literature search system.

**Figure 2:**
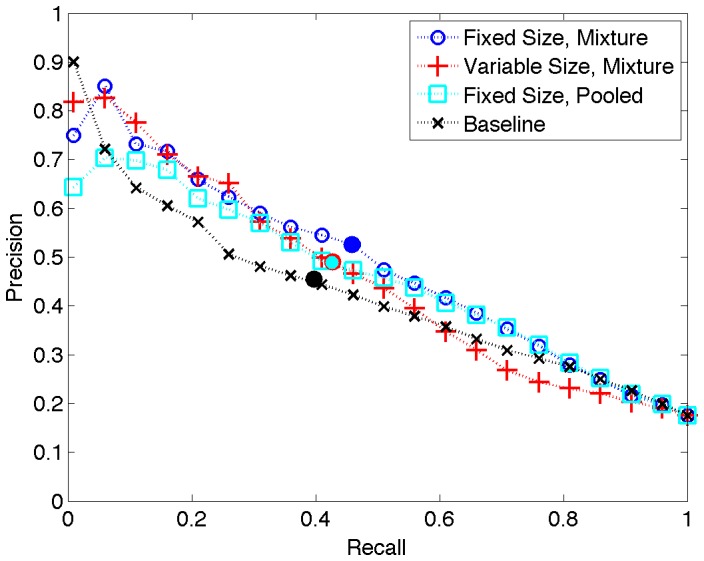
Recall-precision curves for three LMs and the baseline. The (Fixed Size, Mixture) model is our CompleteLM. The filled circles denote locations of the 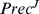 points.

The work presented here extends previous work of our group on linking abstract sentences with figures [1] in several significant ways, and makes important contributions to our understanding of this problem. The present system uses supervised learning approaches while previous methods are unsupervised. We introduce probabilistic language models, position models, and HMM and CRF linkage flow models for this task. We evaluate two kinds of figure representations, one based on text in figure captions and the other on text in referencing paragraphs. A new evaluation measure based on the average number of saved clicks is introduced. Finally, the accuracy of predictions is significantly improved.

Our system is fully implemented and contains over 150,000 open access full-text biomedical articles that can be accessed at http://figureitout.askhermes.org.

### Related Work

In this section we discuss relationships to previous work in four areas: text-based literature search systems, classification and search methods for images in documents, textual entailment, and summarization.

A number of medical and biological text-based literature search systems have been constructed. These include systems that respond to users’ queries, such as PubMed and AskHERMES [4] for medical literature. Textpresso [5] was originally designed to assist biological database curation but also functions as an information retrieval system. Arrowsmith [6] helps biologists formulate hypotheses through text mining of two topics, such as a drug and an adverse event. Other systems attempt to find specific kinds of information. For example, GeneWays [7] extracts molecular interactions related to pathways identified in the literature and iHOP [8] identifies sentences that relate two genes. Additionally, there are numerous annotated databases – for example, the Gene Ontology annotation [9] the mouse Genome Database [10], SWISSPORT, OMIM [11], and BIND [12] – that provide different levels of annotated literature information about genes and molecular interactions. See the review article [13] for other information systems.

In addition to text, the importance of biomedical figures and images for document classification and retrieval has been recognized. The earliest image mining system is the Subcellular Location Image Finder (SLIF) system [14]–[18] which extracts and analyzes fluorescence microscope images from biomedical full-text articles. Other studies have looked at applying supervised machine-learning algorithms for image categorization using flat [19] and hierarchical [20] classification schemes. These methods showed that image classification benefits document classification. Besides the image content itself, associated text has been shown to be important for image mining. Caption words, for example, can improve image classification [19]. BioText searches biomedical images on the basis of image captions [21], [22], and Yale image finder [23] searches images on the basis of title, abstract, image caption, and the text appearing in an image. More recently, an approach has investigated using figure-associated text for automatically ranking figures by their importance [24]. While these methods utilize text for different tasks, they do not automatically associate images or the figures that contain them with specific document text.

Our approaches of associating figures with text are also related to the problem of textual entailment [25], a task that has application to numerous higher-level problems including passage retrieval, machine translation, paraphrasing, summarization, and question answering. The PASCAL Network of Excellence Recognizing Textual Entailment (RTE) challenge task is to recognize whether two text strings can be semantically inferred (entailed) from each other. Thus, a body of text is said to “entail” a hypothesis text if the body of text implies that the hypothesis is true. Our task is similar in that the aim is to determine whether or not one string (a sentence in the abstract) is associated with another string (the text of a figure). The RTE task does not directly apply to the linkage between figures and text as the relationships between linked abstract sentences and figures is generally much weaker than entailment.

Lastly, we find similarities with the computational summarization work of Jing and McKeown [26]. They learn a summarization system from a training set consisting of human-written summary sentences in which words in the summaries are mapped to words in the original article. Their summarization approach, which assumes human summaries are created via a cut-and-paste process, uses two heuristic rules: (1) human summaries are more likely to use whole phrases than single, isolated words, and (2) humans are more likely to merge nearby sentences into a single sentence than combine sentences that are far apart. They model these tendencies with HMMs. These rules parallel patterns of associations between figures and abstract sentences that we represent with HMMs and CRFs. A key difference between these two tasks, however, is that while for summarization Jing and McKeown [26] permit only one-to-one associations between words in summary sentences and words in the original, we allow more general one to many, many to one, and many to many associations between figures and sentences. In this way then, our application represents a more challenging task.

## Results and Discussion

We conducted experiments on a corpus of 114 manually annotated biomedical articles to empirically evaluate our approach to predicting linkages between abstract sentences and figures. Our experiments involve training models and making predictions from a progressively increasing number of evidence types. First, we consider only text, and evaluate predictions of our language models (LMs). Next, we add position evidence, and evaluate predictions of combined LMs and distance models (LM+DM). Last, we add (inferred) linkage evidence, and evaluate predictions of our hidden Markov model (HMM) and conditional random field (CRF) methods.

We designed our experiments to test several statistical hypotheses. Each experiment was evaluated using up to 3 performance metrics commonly used in information retrieval, as well as an application specfic performance value (“clicks”). For each test the null hypothesis is that two competing approaches have the same mean measure (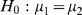) and the alternative hypothesis is 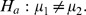 We report 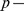
values (the probability of the observed data under 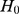) in all cases where 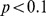) The three hypotheses were:

1. The quality of predictions of our complete language model (CompleteLM) exceeds those of the state-of-the-art approach, which uses only text.
2. Both linkage and positional features are predictive of abstract sentence/figure linkage and are complementary to text.
3. The predictive performance of our HMM and CRF methods, which integrate text, linkage, and positional features exceeds the performance of the state-of-the-art approach.

Our empirical results support each of these hypotheses.

We measure performance using standard measures: precision is the fraction of linked figures that are correctly identified by a system, recall is the fraction of figures linked by a system that are truly linked figures, AROC is the area under the curve defining the false-positive rate as a function of recall, and F1 is the geometric mean of precision and recall.

We also use a new measure that we call “clicks”. This is not actual clicks by a designated user but a mathematical model meant to estimate the savings over a user reading an abstract sentence and then selecting figures sequentially, looking for supporting information for the sentence. Our model assumes that figures are selected in order until the set of figures relevant to a given sentence is found. This may be an overestimate (if the user has visual clues or has already clicked on some of these figures for a previous sentence), or an underestimate (if the user clicks on all sentences, just to be sure, or clicks on the back button) but it does provide consistent criteria for evaluating methods. More precisely, for any sentence, we assume that if Figure 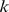 is the last figure (truly) linked to that sentence, a user without our system would click 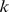 times to retrieve the relevant figures, accessing the figures sequentially until obtaining the desired information. If our system scores all of the relevant figures for the sentence within its top 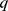 choices, then our user would click 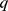 times, and the number of clicks saved would be 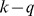. We define “clicks” to be this difference, averaged over all sentences in the set of abstracts. “Clicks” thus represents the average reduction in the number of mouse clicks needed by a user to locate a figure associated with an abstract sentence when the user clicks on figures in the order determined by linkage scores rather than sequentially.

### Results for Language Models

We performed a leave-one-article-out cross-validation experiment to assess the performance of different LM approaches. We evaluated four types of figure-specific models (Caption Only, Referencing Only, Pooled, and Mixture) and two types of background models (Variable Size and Fixed Size) and compare to the current state-of-the-art [1] (Baseline). See the methods section for LM specifics. Since the Mixture figure-specific model considers more text than the other two methods and differentiates between caption and referencing text, and since the Fixed Size background model corrects for a bias that Variable Size has against long sentences, we sometimes refer to the LM (Fixed Size, Mixture) as the CompleteLM. We hypothesize CompleteLM will outperform the other LMs as well as Baseline.

These differing performances are given in Table 1 where the column headers on the per-article side of the the table have an over-bar and subscript 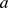 to indicate that the reported values are averages across articles. Broadly, note that the scores for per-article are uniformly higher for all methods and measures than their corresponding scores for the whole-corpus. So, indeed, the methods perform better on articles with fewer links. We will analyze these differences in detail using a permutation test, but first we discuss the results of using differing background models, i.e., differences between top and bottom rows of the table.

**Table 1:** Performance measures of text-only models.

Background	Figure	whole-corpus			per-article		
Model Vocab.	Model Type	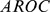	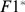	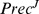	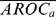	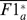	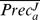
Variable Size	Caption Only	0.66	0.38	0.39	0.69	0.53	0.40
Variable Size	Referencing Only	0.67	0.38	0.36	0.73	0.56	0.44
Variable Size	Pooled	0.74	0.48	0.49	0.76	0.60	0.50
Variable Size	Mixture	0.73	0.47	0.49	0.76	0.61	0.50
Fixed Size	Caption Only	0.71	0.43	0.43	0.74	0.56	0.45
Fixed Size	Referencing Only	0.68	0.39	0.38	0.75	0.58	0.45
Fixed Size	Pooled	0.77	0.49	0.49	0.80	0.63	*0.53
Fixed Size	Mixture	**0.78**	**0.50**	**0.53**	**0.81**	*0.64	**0.54
Baseline		0.75	0.45	0.46	0.80	0.62	0.49

We show performance for our eight language models and the baseline. The first three result columns show overall corpus-wide performance, and the last three result columns show mean performance across articles. The first seven result rows show performance of incomplete LMs, and the eighth result row shows the performance of our CompleteLM, (Fixed Size, Mixture). Our CompleteLM performs best on all measures. Asterisks denote 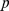-values from paired 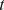-tests comparing each method with Baseline, where * indicates 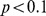 and **indicates 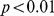.

Reported values in the table are the precisions that arise when the number of predicted linkages is equal to the number of abstract sentences. That is, the precision value 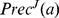 for article 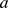 (used in the calculation of 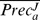 in the per-article method) is the precision for the top scoring 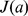 sentence-figure instances, where 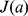 is the number of abstract sentences in article 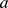. Similarly, 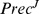 for the whole-corpus case is the precision for the top scoring 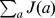 instances. Figure 2 shows whole-corpus recall-precision curves for three LM models and the baseline.

We continue our discussion of results by comparing CompleteLM with Baseline. We observe that the performance of our approach exceeds the baseline on all measures. To estimate significance we conducted paired 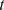-tests for the three per-article measures. The 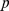-value for 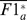 (0.063) nearly indicated significance at the standard 0.05 level, while the 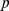-value for the important 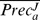 case (0.0071) is significant. Thus, we conclude the expected value of 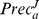 for CompleteLM is larger than for Baseline. Since the 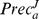 measure, unlike 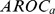 and 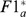, depends only on labels of top scoring instances, the improvement in 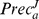 is especially relevant to literature browsing systems, which are likely to provide access to figures for only a few of the highest scoring instances. In the recall-precision curves (Figure 2) we observe that, except for very small recall levels less than 0.05, the CompleteLM curve dominates Baseline up to a recall of 0.8.

We now turn to a comparison of our eight LMs. We look first at the performance of different figure models. For a given background model, the LMs for pairings with Mixture and Pooled figure models have consistently better performance than pairings with both Caption Only and Referencing Only models. Paired 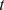-tests on the per-article measures confirm that for all cases these differences are significant (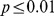). We conclude that our LM approach successfully combines complementary sources of text. Next, we compare our two background models. The Fixed Sized models, which have background vocabulary sizes set to a constant value because of a potential bias against linking long sentences, have better performance than Variable Size models when comparisons are made between pairings with the same figure model. Differences between Fixed Sized and Variable Size background models paired with Mixture figure models are significant (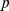-values of paired 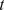-tests 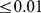) for all three measures.

**Figure 3:**
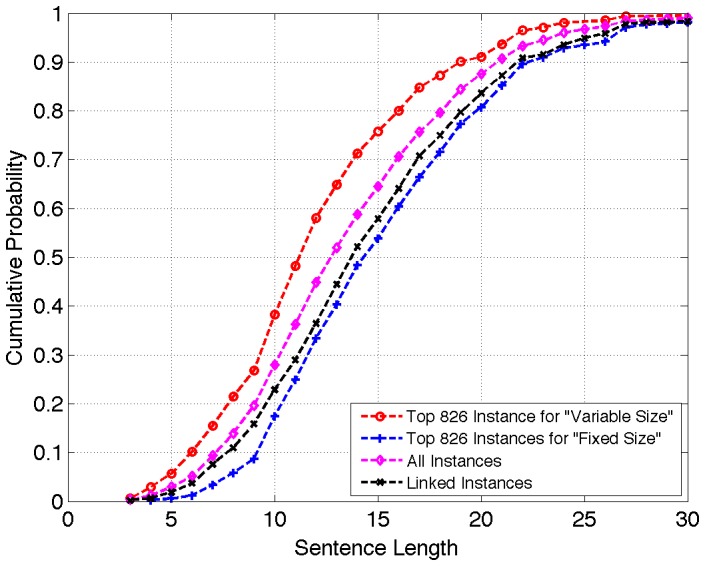
Empirical cumulative distribution functions of sentence length for four collections of instances: all 5406 instances, all 947 linked instances, and the top 826 scoring instances from (Fixed Size, Mixture) and (Variable Size, Mixture) language models.

To investigate a possible bias favoring links to longer sentences we plot in Figure 3 empirical cumulative distribution curves of sentence lengths for four collections of sentence/figure instances: all 5402 instances (magenta line), all 947 linked instances (black line), and the top scoring 826 instances under (Variable Size, Mixture) (blue line) and (Fixed Size, Mixture) (red line) models. We choose 826 because this is the total number of abstract sentences in our corpus, and thus these curves show sentence length distribution at the 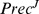 point. The Variable Size method prefers linking short sentences rather than long sentences (gap between the red and black curves for a given sentence length). For example, although only 58% of linked instances have sentences with ten or fewer terms, 76% of all high-scoring instances under the Variable Size approach have sentences at least this short. The Fixed Size approach eliminates this bias, especially for sentences longer than 10 terms. Interestingly, there appears to be an actual preference *for* longer sentences to be linked as seen by comparing the magenta “All Instances” curve to the black “Linked Instances” curve. This may be reflective of a positive correlation between sentence length and information content.

Lastly, we look in more detail at uniformly higher performance in the per-article metrics by setting up an experiment to see if the differences are due to in-homogeneity of the data. That is, do some articles have language modelling scores that make linking sentences with figures easier? This may be due to an author’s style or the topic being discussed. We explore this by employing a permutation test. We hold model type fixed and compare performance values calculated using the whole-corpus method with their per-article counterparts (that is, we compare values within rows of Table 1). We observe that for all three measures and all models the per-article performance value is larger than the whole-corpus value. While due to the way 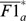 is calculated, we expect the larger per-article values for this measure, there is no calculation bias for area under the ROC curve and precision.

**Figure 4:**
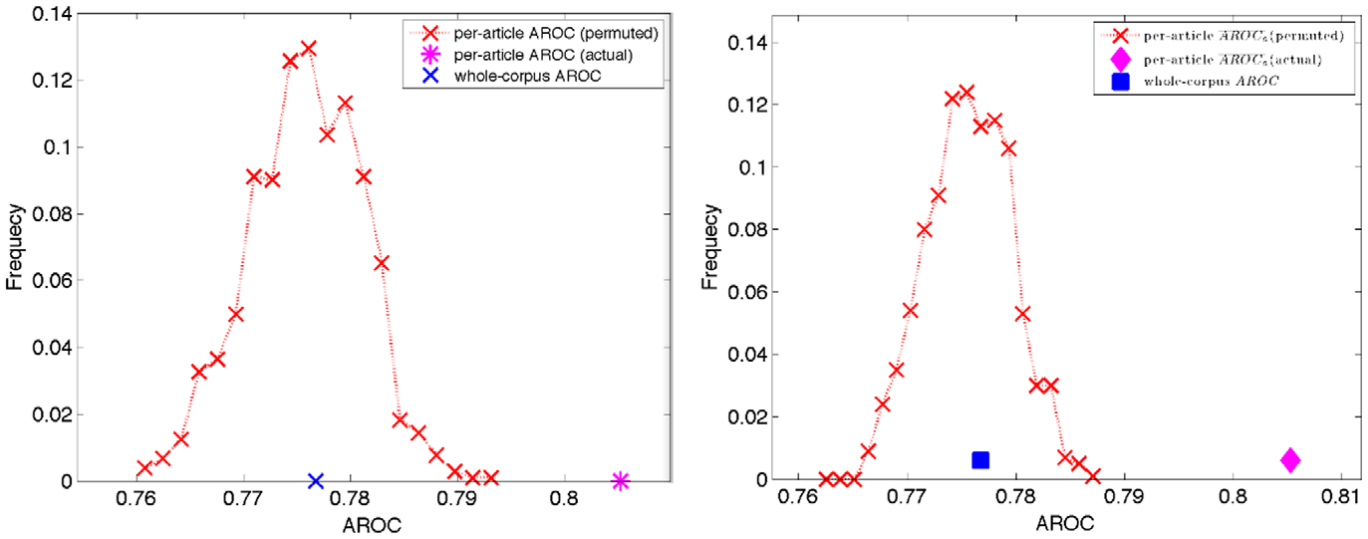
Results of permutation tests showing article-effects on two performance measures: area under the ROC curve (left) and precision (right). Blue and magenta points show actual performance values for the CompleteLM model calculated with the whole-corpus and per-article methods, respectively. The red-line shows a normalized histogram of per-article performance for 1000 random permutations of the associations between articles and abstract sentence/figure instances.

The permutation test shuffled the associations between articles and sentence/figure instances, keeping the number of linked instances associated with each article fixed. Although whole-corpus performance values do not depend on article assignments, per-article performance values do. For each of 1000 permutations we computed 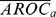 and 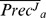 from linkage scores of CompleteLM using the shuffled article associations. Figure 4 shows normalized histograms of observed performance values from the permutation test along with actual whole-corpus and per-article values. Although there is an article effect for both measures, it is clearest for AROC. On this measure, while the whole-corpus 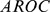 value of 0.777 was near the median (exceeding the per-article value in 485 of the 1000 permutations) the actual per-article 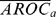 of 0.805 was substantially larger than any of the permuted values. On precision the whole-corpus 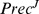 value was larger than 904 of the permuted values, and the actual per-article 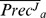 was larger than 999 of the 1000 permuted values. One consequence of the observed article effect is that since in a literature browsing system linkage scores are only considered one article at a time, whole-corpus performance measures will underestimate system performance in practice. A second and more important consequence is that a single, fixed threshold on linkage score separating positive and negative predictions is not appropriate. It will be too permissive for articles with score-increasing effects, and conversely too restrictive for articles with score-reducing effects.

### Results for Combined Text and Non-text Models

In this section we evaluate approaches to linkage prediction that utilize both text and non-text features. We consider two kinds of non-text features. Values of *positional* features are based on the relative positions of the sentence within the abstract and the figure within the article, and values of *linkage* features are derived from linkage patterns of other instances in the same article. While the values of positional features are always observable, the values of linkage features are generally not observable when predictions are needed. Although the linkage values are hidden, since the hidden Markov model (HMM) and conditional random field (CRF) approaches collectively classify all of an article’s instances simultaneously, inferred values of linkage features can inform their predictions. We first evaluate non-text features individually, and then in combination.

#### Information gain of non-text features

Table 2 shows the percent information gain (% gain) of non-text features. For comparison, we also show the % gain of the text-based CompleteLM model’s linkage scores (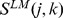, see methods). A feature’s % gain indicates how predictive of linkage that feature is in isolation. It ranges from 0% (not predictive) to 100% (completely predictive). It is not surprising that the % gain of CompleteLM scores (16.30%) is, by a wide margin, the largest, as these scores come from models of numerous text features while the other % gain values in the table are for individual features. After CompleteLM, the next three features form a well separated group with similar % gains. This group comprises two linkage features, EdgesCrossed (7.82%) and FigureDegree (7.37%), along with Distance (7.48%), a positional feature. The relatively high gain of Distance agrees with previous work [1] where we found that predictions based on text and Distance are more accurate than predictions of text-only models. The Distance feature is, however, the only non-text feature previously used for abstract sentence/figure linkage prediction. Therefore, the present work represents the first time the other features in Table 2 have been considered for this task.

**Table 2:** Percent information gain of non-text features.

Feature Name	Feature Kind	% Gain
CompleteLM scores	–	*16.30
EdgesCrossed	linkage	*7.82
Distance	position	*7.48
FigureDegree	linkage	*7.37
PreviousFigure	linkage	*4.55
PreviousSentAndFig	linkage	*4.01
PreviousSentence	linkage	*1.78
InitialSentence	position	*1.57
SentenceDegree	linkage	*1.51
LastSentence	position	0.08
InitialFigure	position	0.00
LastFigure	position	0.00

For comparison with text features we also show the gain of CompleteLM scores. Asterisks indicate features whose % gain significantly differs from 0.0 (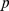-value of permutation test 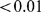).

Other than Distance, Initial Sentence, with a modest gain of 1.78%, is the only positional feature with % gain significantly different from 0.0. All linkage features, on the other hand, have statistically significant % gain values. Indeed, four linkage features have gains exceeding 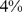. If appropriately modeled, these linkage features may lead to more accurate predictions of abstract sentence/figure associations. Incorporating them into a model, however, is challenging since linkage feature values are unobserved at prediction, and therefore approaches that predict linkages of each instance independently are unable to use linkage features. In fact, a key motivation behind our HMM and CRF approaches was to utilize their collective classification properties to model linkage features.

#### Evaluation of linkage predictions

To evaluate models of text and non-text features, we performed leave-one-article-out cross-validation experiments similar to those we used to evaluate language models. We look at three approaches to modeling text and non-text. Our CompleteLM+DM approach combines CompleteLM and distance model (DM) scores (Equation 13). Like the LM approaches, it predicts linkages independently for each sentence/figure pair. In contrast, our other two approaches, HMMs and CRFs, make collective predictions, and moreover utilize both positional and linkage non-text features. We evaluate both the sentences-in-states (SIS) and figures-in-states (FIS) HMM and CRF variants. We compare predictions of our models that merge text and non-text features to predictions of models that consider only distance (DM), only text (CompleteLM), and two baselines: the text-only Baseline described above and a combined text and distance method used in a previous study [1]. This text and distance baseline – called 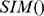 by its authors, but which for consistency we refer to as Baseline+DM – represents the current state-of-the-art, and is currently the most accurate method for predicting abstract sentence/figure linkage. As above, we use the 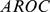, 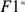 and 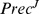 performance measures calculated corpus-wide and per-article. Additionally, we report per-article values of clicks, labeled 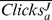.

**Figure 5:**
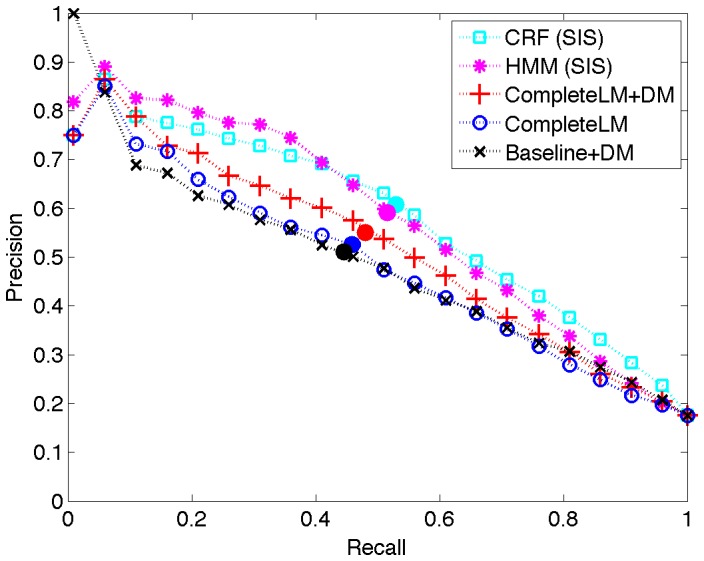
Whole-corpus recall-precision curves. The solid dots indicate the recall-precision point at 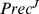, when the number of predicted linked instances is equal to the total number of abstract sentences in the corpus.

Table 3 shows the performance of various models, and Figure 5 shows whole-corpus recall-precision curves for a subset of models. CRF (SIS), our top performing model, has the highest performance on all measures. Paired 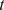-tests indicate that differences between CRF (SIS) and Baseline+DM for all per-article measures are statistically significant (all 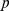-values 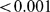). From the recall-precision curves in Figure 5 we see that, except for recall levels 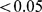, the CRF (SIS) curve dominates the Baseline+DM curve. Therefore, we conclude that CRF (SIS) represents a significant improvement over the state-of-the-art for predicting linkages between abstract sentences and figures.

**Table 3:** Performance values for models that use combinations of text, positional and linkage features.

				whole-corpus			per-article			
Method	T	P	L	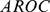	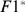	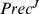	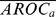	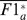	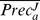	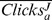
DM		✓		0.67	0.39	0.26	0.70	0.46	0.31	0.97
CompleteLM	✓			0.78	0.50	0.53	0.81	0.64	0.54	1.52
CompleteLM+DM	✓	✓		0.80	0.53	0.55	0.82	0.67	0.58	1.65
HMM (FIS)	✓	✓	✓	0.80	0.54	0.57	0.81	0.66	0.57	1.74
CRF (FIS)	✓	✓	✓	0.81	0.54	0.56	0.83	0.67	0.58	1.75
HMM (SIS)	✓	✓	✓	0.82	0.56	0.59	0.84	*0.68	*0.60	1.57
CRF (SIS)	✓	✓	✓	**0.84**	**0.57**	**0.61**	**0.86	**0.69	**0.62	**1.82
Baseline	✓			0.75	0.45	0.46	0.80	0.62	0.49	1.49
Baseline+DM	✓	✓		0.79	0.49	0.51	0.83	0.65	0.57	1.65

Values calculated across the whole-corpus as well as per-article averages are shown. Each row gives the performance measures for one model. The **T**, **P** and **L** columns indicate with a 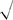 those models that use text, positional, and linkage features, respectively. The sentences-in-states CRF approach, CRF (SIS), has the top performance on all measures. Asterisks denote 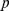-values (*denotes 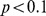 and **denotes 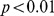) from paired 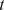-tests comparing the per-article performance of our methods to those of Baseline+DM, the current state-of-the-art.

Comparing the CRF and HMM methods, we observe that CRFs usually, but not always, outperform HMMs for the same variant (either SIS or FIS). The HMM (SIS) has higher precision than CRF (SIS) for recall levels below about 0.4 while CRF (SIS) has the higher precision at higher recall levels. However, when we compare the SIS and FIS constructions we see that HMM (SIS), the least performing SIS construction, outperforms both FIS approaches. Hence, it appears model variant (SIS or FIS) has more effect on performance than model type (HMM or CRF). Even so, the differences between CRF (SIS) and HMM (SIS) are significant (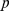-value 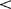 0.05) for 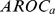 and 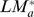 (but not 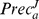). Since aspects of the FigureDegree feature are captured by the SIS CRF but not the FIS variant, the superiority of the performance of SIS over FIS agrees with the relative information gain values (Table 2) of FigureDegree (7.37%) and SentenceDegree (1.51%). To better understand the performance differences between CRF (SIS) and CRF (FIS), we compared articles for which CRF (SIS) had larger 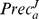 score to those where CRF (FIS) had the higher score. Articles won by CRF (FIS) had on average of 1.11 fewer abstract sentences than articles won by CRF (SIS). A permutation test reveals that this 1.11 sentence difference is statistically significant. This suggests a suite of models approach, where the model applied to linkage prediction on a given article depends on the number of abstract sentences or other observable article properties, may be effective.

The overall trend evident in the performance measures of Table 3 and the recall-precision curves in Figure 5 is that performance increases as more types of features are utilized. Of the models that use a single class of features, those that use text only are clearly superior to the DM approach. Combining DM with text models gives a substantial performance boost, most markedly for Baseline+DM versus Baseline. We see another performance bump for models that incorporate linkage features. Thus, we conclude that text, positional and linkage features are complementary for linkage prediction, and that our approaches successfully integrate these diverse types of evidence.

### Results for Human Annotators

We invited authors of a disjoint set of 49 additional PNAS articles to provide annotations of abstract sentence/figure associations for their articles, and to evaluate a prototype of our online article browsing system on their articles. We subsequently asked authors to complete a short four question usability survey. A total of 21 authors participated for a response rate of 43%. Further, we asked three bio-medical researchers who are not authors of any of these articles to provide additional annotations from which we obtained linkage annotations for 14 of these articles.

The 14 articles annotated by both authors and non-authors contain a total of 420 abstract sentence/figure instances. Table 4(a) shows the contingency table for the linkage annotations of these instances. Authors and non-authors have a related concept of sentence/figure association (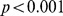 for 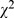 test on independence of counts in Table 4). Authors and non-authors agree on linkage status on 81% of instances, and inter-annotator agreement as measured by Cohen’s 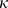 is 0.47. The concept of association, however, is not precise as non-authors and authors disagree 19% of the time. It is interesting that non-authors, with a 27% linked rate, appear to have a significantly more liberal notion of association than authors, who identify only 17% of instances as being linked.

**Table 4:** Results for 14 articles with human annotations provided by both authors and non-authors, and computational predictions provided by the CRF (SIS) model.

(a)		Authors		
		Non-linked	linked	Total
Non-Authors	Non-linked	283	22	305
	Linked	58	57	115
	Total	341	79	420

(a) Contingency table of human annotations.

(b) Per-article average recall, precision and F1 score of non-author human annotations and computational predictions using author annotations as ground truth.

Besides estimating inter-annotator agreement we can use non-author annotations to compare the computational predictions of our models with human predictions. Using author annotations as ground truth, we compare the performance of linkage predictions made by humans (*i.e.,* non-authors) with computational predictions. Using the CRF (SIS) model trained from author annotations for the 114 articles used above, we predicted linkages for the 14 articles that have both author and non-author annotations. For an article with 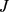 abstract sentences we predict that the top scoring 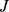 instances are linked and that the other instances are not linked. Thus, the CRF (SIS) row of Table 4 are 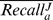, 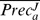 and 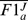 values. We point out that there is a difference between the 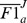 measure in Table 4 (average of 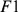 calculated at one pre-determined point in each article) and the 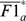 measure in Tables 1 and 3 (average of maximum 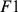 value in each article). While human annotators have higher performance values on all measures (none of these differences are statistically significant), the performance of CRF (SIS) is competitive with that of the non-author humans. On 9 of the 14 articles the human had the higher 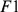 score, while on the other five articles CRF (SIS) had higher 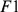.

Table 5 shows the pilot survey questions and average response values. We observe that users tend to have a positive view of the accuracy and usability of the prototype system. Interestingly, there is a significant positive correlation between an author’s score for Q2 (“How useful are current figure-sentence associations?”) and the 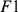 score of the system predictions for their article (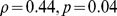). Similar correlations were observed for other questions. From these results we conclude that methods for making more accurate predictions of sentence/figure associations, including the computational approaches we describe in this article, will lead to more usable online literature browsing systems.

**Table 5:** Survey questions and average response values.

**Q1:**	How accurate are the figure-sentence associations? (3.76)
**Q2:**	How useful are the current figure-sentence associations? (3.62)
**Q3:**	If the system is implemented, how eager will you be to use it? (3.57)
**Q4:**	Do you like the interface design? (4.10)

Values range from 1 (not at all) to 5 (very).

#### Conclusion

We have described methods for computationally identifying associations between sentences in the abstract of a scientific article and figures (and tables) in the article body. We use supervised methods for learning. Our models use three types of evidence to predict whether or not an abstract sentence is linked with a figure: text (in the abstract sentence, figure caption, and passages that refer to the figure), the relative positions of the abstract sentence and figure, and patterns of inferred associations for other sentence/figure pairs in the article.

Each type of evidence has predictive value. Our experimental evaluation showed that models that use all evidence types are more accurate than models that use only one or two types of evidence. Our best performing models, based on conditional random fields (CRFs) [3], achieve a macro-average F1 score of 0.69. The area under its ROC curve is 0.86. These performance measures represent a statistically significant improvement on the state-of-the-art for this task, an unsupervised approach developed earlier [1]. Moreover, disagreement of human annotators on linkage status is nearly as common as prediction errors of our system.

We observed that the use of a language model significantly improved the results of previous work, where a TFIDF cosine similarity was used. Once a larger data set is collected and more detailed user feedback is assembled, a natural area of future exploration is more sophisticated language models. For example, the use of word bigram models, smoothing based on related clusters of articles, and divergence metrics such as Jensen-Shannon are all possible extensions of this work [27].

Automatic methods for predicting linkages between abstract sentences and figures are important for the development of the next generation of literature search and browsing systems. A user study showed that users find the figure browsing features supported by our linkage predictions to be helpful. We have incorporated linkage predictions into our system (http://FigureItOut.askHERMES.org).

## Methods

### Data and Features

We re-use our collection of 114 full-text biomedical articles (39 from Cell, 29 from EMBO, 30 from the Journal of Biological Chemistry, and 16 PNAS) from our previous study [1]. The authors manually annotated their articles by identifying associations between abstract sentences and figures. The collection has 826 abstract sentences, 741 figures, and 5402 total sentence/figure instances of which 947 (17.5%) are linked. Of the abstract sentences 271 (32.8%) are not linked with any figure, 317 (38.4%) are linked with a single figure, and 238 (28.8%) are linked with multiple figures. And for figures, 91 (12.3%) are not linked with any abstract sentence, 423 (57.1%) are linked with a single sentence, and 227 (30.6%) are linked with multiple sentences. The range of the number of abstract sentences and figures in an article is [3], [13] and [3], [11], respectively.

#### Term vectors

We represent the text content of captions and referencing paragraphs with the “bag-of-words” representation, and for abstract sentences we use the “set of words” representation. The term vector 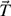 for a sentence or figure has 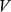 elements, one element for each term in the vocabulary. For figures, 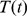 is the number of occurrences of term 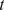, while for sentences it is a binary indicator of the presence (1) or absence (0) of 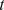.

#### Positional features

In addition to text we also use non-text features. The features naturally divide into two groups, positional features and linkage features. The value of the positional features for sentence 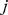 and figure 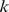 in article 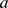 depends on the positions 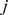, 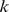 and the total number of abstract sentences (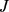) and figures (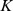) in 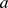. We number sentences and figures sequentially as they appear in the article. So, for example, instance (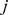,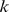) is for the 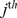 abstract sentence and 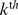 figure in the article.

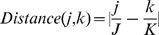
. This feature measures the difference of the relative sentence and figure positions. It is the only non-text feature previously used for predicting sentence/figure linkages.

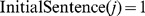
 if 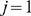 and 0 otherwise.

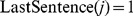
 if 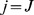 and 0 otherwise.

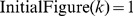
 if 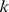 = 1 and 0 otherwise.

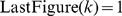
 if 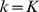 and 0 otherwise.

#### Linkage features

**Figure 6:**
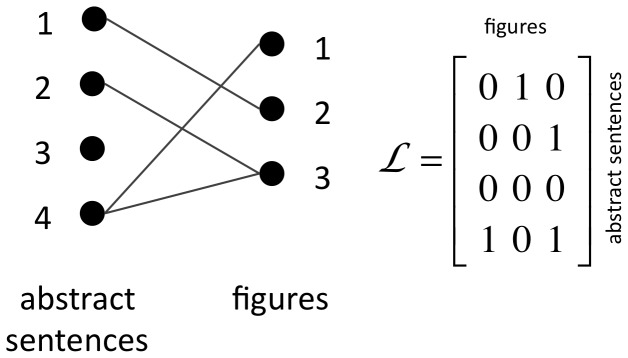
Example graph and linkage matrix representations for an article with four abstract sentences, three figures and four sentence/figure links. Combinations of linkages that induce edges that cross in the graph representation, 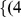
–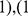
–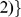 and 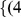
–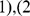
–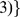 in this example, are less common as they are out of keeping with the observed tendency for consistent relative ordering among linked instances.

We compute the value of linkage features from article-wide linkage patterns. We represent the linkage of an article with the 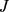-by-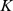 linkage matrix 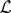, where 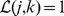 if sentence 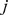 is linked with figure 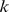, and 0 otherwise. Figure 6 shows an example, which we use in the following six definitions of linkage features.

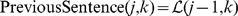
 (undefined when 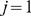). This feature indicates if 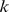 links with the abstract sentence previous to 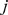. The value of 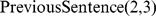 is 0 because sentence 1 and figure 3 are not linked.

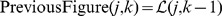
 (undefined when 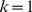). This feature indicates if 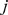 links with the figure previous to 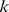. The value of 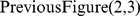 is 0 because sentence 2 and figure 2 are not linked.

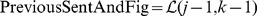
 (undefined if 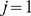 or 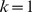). This feature indicates if the previous sentence and figure are linked. The value of 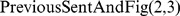 is 1 because sentence 1 and figure 2 are linked.

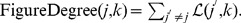
 This feature is the number of sentences (other than 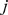) linked with figure 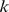. The value of 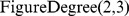 is 1 because sentence 4 and figure 3 are linked.

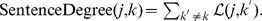
 This feature is the number of figures (other than 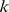) linked with sentence 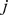. The value of 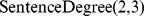 is 0 because sentence 2 does not link with any other figure.
- Edges Crossed(*j*, *k*)  = 

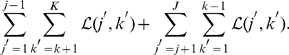



This feature is the number of links inconsistent with the preservation of relative ordering across links. The name EdgesCrossed comes from number of edges that would be crossed by the edge 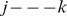 in the graph representation of 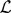. In the example in Figure 6, the value of 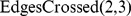 is 1 because the edge 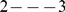 crosses the single edge 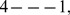 and 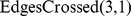 is 2 because the edge 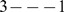 would cross 2 edges.

Since 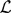 is not observed while predicting, linkage feature values are also hidden. Therefore, these features are not helpful in methods that predict instance linkages independently. The inferred values of linkage features may, however, benefit prediction by techniques like our HMM and CRF approaches.

### Language Models

We model text properties of linked and non-linked instances using probabilistic language models (LM). Our LM approach is motivated by the successful application of similar methods to document retrieval [27]–[29]. For document retrieval the LM approach induces for each document a probability model over all terms in the vocabulary. Then, a document’s relevance to a query is defined as the probability of the query under its model.

Hiemstra’s LM approach [28] uses two kinds of term distributions: a single background distribution 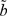 shared by all documents, and a set of document-specific distributions 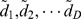 for a 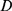-document corpus. The LM represents the probability of query terms for document 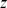 as a mixture of 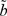 and 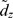. This mixture distribution corresponds to a generative process for constructing query terms for 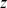 that first randomly selects either 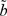 or 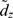 according to the mixing distribution (parameterized by 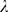) and then samples a term from the chosen distribution. To generate a query with 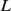 terms, these two steps are repeated 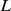 times.

In a similar way we use language models to predict links between abstract sentences and figures by treating abstract sentences as queries and figures as documents. Let 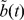 and 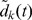 be the probability of term 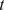 under the background distribution and figure 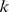
’s distribution respectively. Then, the probability of abstract sentence 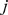 given that it is linked to figure 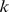 is.
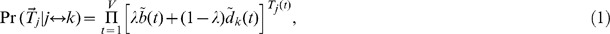
(1)where 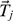 is sentence 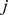
’s length 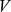 term vector, 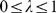 is the mixing proportion for the background distribution, and 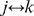 denotes that 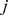 and 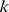 are linked, or equivalently that 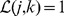. If 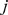 and 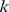 are not linked, the background distribution generates all terms in the sentence:


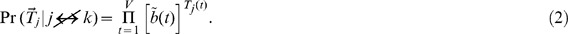
(2)

The LM score matrix 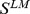 for an article holds the log-odds of the sentence terms given linkage for all instances 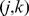. For an article with 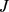 sentences and 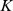 figures, 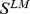 is 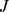-by-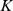 and.


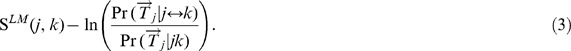
(3)

#### Figure-specific models

A natural and often-used representation of the document-specific term distribution 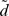 is a multinomial distribution where each probability 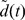 has its own parameter. Parameter estimation for the multinomial model typically treats all occurrences of 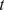 in the document equally, and sets 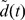 to its frequency in the document. We consider multinomial representations, but we also use a representation that distinguishes caption terms from terms in referencing paragraphs.

Since in our approach to the linkage prediction task, figures play a role analogous to documents, to apply the multinomial approach we need to determine which terms represent a figure. Candidate term sources include terms in the figure’s caption as well as terms in the article body close to figure references. We consider three sources: caption terms (Caption Only), terms in referencing paragraphs (Referencing Only) and the combination of terms in either the caption or a referencing paragraph (Pooled).

Let 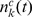 be the number of occurrences of term 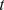 in figure 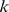
’s caption and 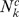 be the total number of terms in the caption. We similarly define 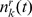 and 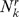 for figure 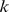
’s referencing paragraphs. The probabilities 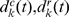 and 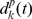 of term 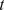 in the Caption Only, Referencing Only, and Pooled representations are simply its frequency in each collection:
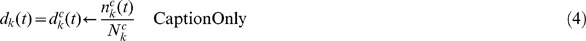
(4)

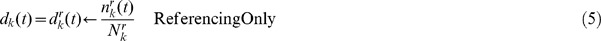
(5)

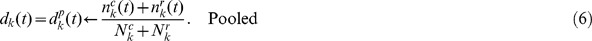
(6)


Note that we do not use pseudo-counts here, as smoothing is unnecessary because the background distribution 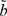 is used for terms that have zero probability in the figure-specific model.

Although the Pooled method has the advantage of including text from multiple sources, it is limited in that it ignores term origin even though there may be meaningful differences between terms in captions and referencing paragraphs. For example, while text in referencing paragraphs can discuss topics unrelated to the figure, caption content nearly always relates to the figure. Our final figure-specific term distribution, which we call Mixture, distinguishes between caption terms and referencing paragraph terms. In the Mixture approach we represent 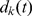 itself as a mixture of the Caption Only and Referencing Only distributions,
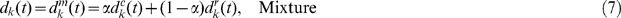
(7)where 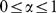 is the mixing proportion for the caption distribution.

#### Background models

We consider two approaches to setting the background distribution of article 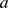. One approach pools all terms present in abstract sentences, figure captions and referencing paragraphs in 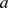, and sets background probabilities to the smoothed term frequencies,
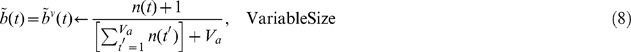
(8)where 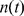 is the count of term 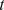 in the pooled collection and 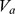 is the number of distinct terms in article 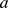
’s sentence/caption/referencing paragraph pool. Since the vocabulary size 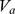 - which depends on the number of distinct terms in abstract sentences, captions and referencing paragraphs - varies from article to article, we call this approach to setting the background distribution VariableSize. Because of finite sampling, however, Equation 8 may lead to biases that favor linking short sentences and against linking long sentences. This bias arises because the probabilities 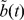 of terms present in the pooled collection set according to Equation 8 are too large. The 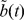 tend to be overestimates because, since the pooled collection is unlikely to contain all terms in the vocabulary, any term not in the pool has (an implicit) background probability of zero. Therefore, the probabilities of the absent terms are underestimated, and their true probability mass is distributed among the probabilities of the present terms. From Equations 1-3 it can be seen that overestimates of 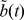 cause a corresponding overestimate of 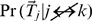 (and thus underestimation of 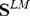) that increases with sentence length.

To correct for the bias in VariableSize, we consider an alternative approach to estimating 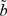 that uses a fixed vocabulary size of 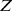 terms in all articles. We call this approach FixedSize. We use a pseudo-count of 1 for all terms, and set the background probability for term 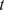 to.
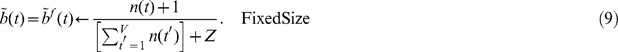
(9)We describe below how we set 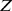 from training sets.

#### Learning language models

We evaluate our LMs with leave-one-article-out cross-validation experiments. Our experiments evaluate each of the eight kinds of LMs: one LM for each pairing of a figure-specific-model (four kinds) with a background model (two kinds). For the cross-validation fold in which article 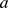 is in the test set, since the background and figure-specific distributions for 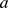 are set from only the terms in 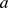 and not any linkages, the parameters in 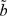 and the 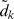 do not depend on the training set of annotated articles. We do, however, use training sets to estimate our other LM parameters: the mixing proportions 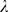 and 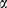, and the fixed-vocabulary size 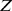.

We estimate separate parameter values for each LM. We first set 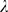
’s for the (VariableSize, Caption Only), (VariableSize, Referencing Only) and (VariableSize, Pooled) approaches. We search over 99 values of 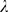 equally spaced from 0.01 to 0.99, and set 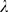 to the value that maximizes the mean 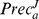 on the training set. Next, we set 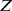 for the (FixedSize, Caption Only), (FixedSize, Referencing Only) and (FixedSize, Pooled) models. For each figure-specific-model we temporarily set 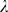 equal to the value just set for its pairing with VariableSize, and then estimate 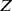 by the value that minimizes the absolute value of the Pearson correlation between sentence length and 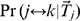 for all training instances 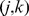. With 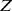 set, we then estimate 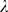 for these three LMs as we do above, by the value that minimizes the mean 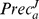. Lastly, we set parameters of LMs with Pooled figure-specific-models. We set 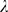 and 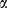 for (VariableSize, Pooled) with a method similar to the method we use to set 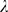 for the other VariableSize models, though now we compute 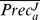 for joint 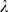, 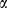 settings. So as to maximize the diversity of our parameter search, we define 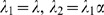, 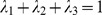, and conduct our search on 120 evenly spaced points on the standard 2-simplex: 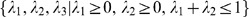. Finally, we set 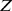, 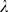 and 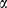 for (FixedSize, Pooled) analogous to the method we use above to set 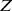 and 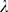 for VariableSize models. We first set 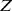 by minimizing correlation between sentence length and score, and next set 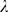 and 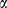 by search on the 2-simplex.

### Distance Model

We begin our description of non-text models with models of the Distance feature. We consider distance models (DMs) because it has been shown previously [1] that the relative positions of an abstract sentence and figure correlate with linkage status. For example, a sentence near the beginning of an abstract is more likely to be linked with a figure near the beginning of an article than with a figure at the end of the article.

We learn models of discretized values of the 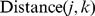 feature for linked and non-linked instances. Let 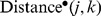 denote the bin of 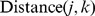 where we have ten bins, and we place bin boundaries so that an approximately equal number of points falls in each bin. We set the bin probability of bin 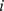 in the DM of linked instances to the Laplace smoothed fraction of linked instances with 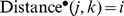,
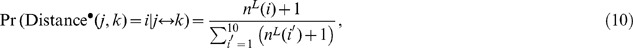
(10)where 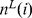 is the number of linked training set instances in bin 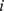. We set bin probabilities for the DM of non-linked instances in a similar way,



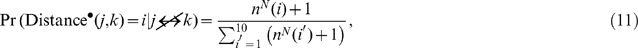
(11)where 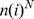 is the number of non-linked training set instances in bin 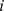.

The distance model score matrix 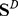 for an article holds the DM log-odds of the article’s sentence/figure instances,


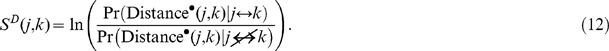
(12)

We construct scores of a combined language and distance model by adding scores,



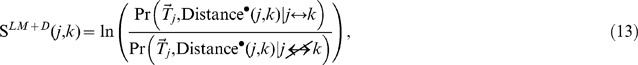
(13)

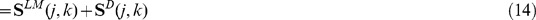
(14)where 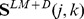 is the combined LM and DM score for sentence 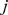 and figure 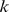, and Equation 14 follows from Equations 3 and 12 under the assumption that terms and distances are conditionally independent given linkage status.

### Hidden Markov Models

In addition to patterns of the Distance feature for individual instances, linkage patterns across instances also have tendencies. For example, given two linked instances, 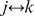 and 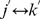, from the same article, if 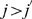, then it is also likely that 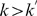. We model these kinds of linkage-flow patterns flow using hidden Markov models (HMMs) [2] and Conditional random fields (CRFs) [3], two kinds of probabilistic models widely used for representing structure in sequential problems. Since flow tendencies indicate preferences for linkage patterns that are, to a certain extent, independent of text, we do not want to ignore text. Both HMMs and CRFs are convenient in this regard as they provide a natural way to model both kinds of evidence. We model flow with state transition probabilities learned from a training corpus, and we model text with emission probabilities derived from the scores of learned language models. We first describe our HMM approach, and then we describe our related CRF approach.

**Figure 7:**
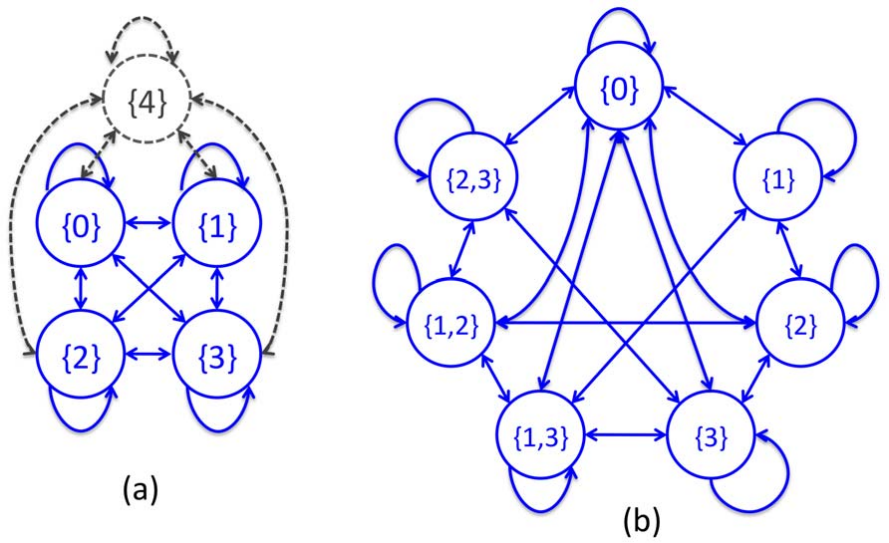
Example HMM (a) and CRF (b) state transition diagrams using the sentences-in-states construction. (a) States and transitions for the base HMM for a corpus where the maximum number of abstract sentences in an article (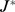) is 4. The states and transitions in sold blue are part of the derived HMM for an article with 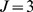 sentences. (b) CRF states and transitions for an article with 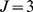 sentences and where the maximum number of sentences per figure (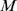) is 2.

We consider two HMM constructions: “sentences in states” (SIS) and “figures in states” (FIS). Our description is in terms of the SIS construction, but FIS can be understood by swapping ‘sentence’ with ‘figure’ in the description. Under the SIS construction, an article with 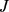 sentences and 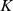 figures has an HMM with 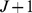 states, 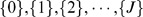, and the length 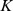 observation sequence (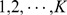). State 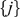 is associated with abstract sentence 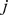, and the non-linked state 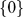 is not associated with any sentence. We have a transition between every pair of states. Figure 7(a) shows an example HMM.

We associate linkage-predictions with state-sequence paths. For example, for an article with 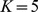 figures and 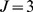 sentences, the path 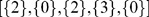 asserts that Figure 1 links with Sentence 2, Figure 2 does not link to any sentence, Figure 3 also links with Sentence 2, Figure 4 links with Sentence 3, and Figure 5 does not link with any sentence. With the SIS construction, a single path can link a sentence to any number of figures 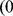 to 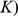, while a figure can only link with 0 or 1 sentences. Our CRF approach relaxes this constraint and permits figures to link with multiple sentences.

As articles have different numbers of abstract sentences, their HMMs have different numbers of states. Our approach to this variation is to learn transition probabilities for a single base HMM structure with 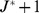 states, where 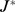 is the maximum number of abstract sentences in any article of the training corpus. Then, for an article with 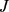 abstract sentences, we construct a 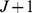-state HMM to predict linkages. The transition probabilities of the derived HMM come from the base structure, while the emission probabilities are based on language model scores.

Training the base HMM structure involves estimating the entries of its 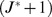-by-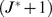 transition matrix 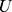. The value 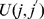 is the probability for the transition from 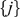 to 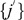. In other words, 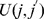 is the probability of 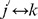 given 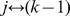, for all 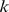. Here, we include unlinked figures in our notation by defining 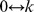 to mean that 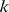 is unlinked. We estimate 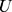 from the training corpus’s transition counts matrix 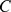, where 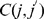 is the number of times that 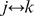 and 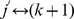 in the training set:
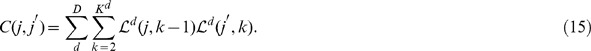
(15)


Here 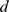 indexes training set documents, and 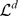 is the linkage matrix for training document 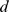. We set the 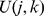 to their MAP estimate using Dirichlet priors with hyperparameters set to 1.0:
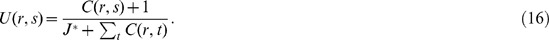
(16)


We create the derived HMM with states (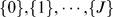) by extracting the corresponding states and transitions between them from the base structure, and re-normalizing transition probabilities so that the sum of the outgoing probabilities from any state is 1.0. If a test-set article happens to have more abstract sentences than any article in the training corpus, we create its derived HMM by adding states to the base structure. Then, we also add transitions so that the derived HMM is fully connected, assign small probabilities to the new transitions, and re-normalize.

We now describe how we set the emission probabilities of the derived HMMs to model text. Since the observation for an article with 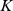 figures is the ordered sequence (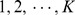), state 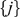 emits symbol 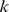 only if 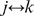. We thus set 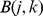, the emission probability for symbol 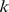 in state 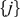, based on the textual coherence between abstract sentence 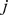 and figure 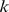. While our language models, of course, are designed to do just this, setting the 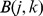 directly from LM probabilities gives poor performance. The primary problem with this approach is that the LM probabilities are not well calibrated. As with other naive Bayes-like models, the posteriors of our LMs tend to be extreme, that is, very close to zero or one. Although models with uncalibrated probabilities often perform well in classification tasks [30], when such models are used as components within a larger model like an HMM predictions can be poor as inference in this case involves reasoning with many uncalibrated probabilities. Therefore, we represent the emission probabilities using Gaussian models of LM scores.

We learn one Gaussian model of LM scores for linked instances and another for non-linked instances. As this approach applies to the scores 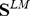 of any language model or the scores 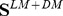 of the combined language and distance model, for generality in our description we denote scores by 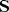 as the computations are the same in all cases. From the training corpus we calculate the sample mean and variance of 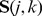 over all linked (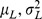) and non-linked (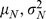) instances, and use these parameters to define Gaussian distributions for 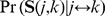 and 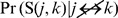 .

Let 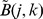 be the joint probability under these models of all scores for figure 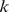: 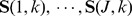, given that it links only with sentence 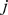, 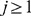:



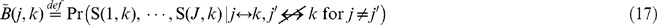
(17)

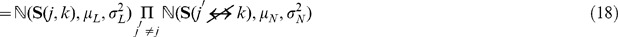
(18)where 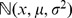 denotes the value of the probability density function for the Gaussian with parameters 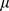 and 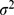 at 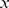. Equation 18 assumes the elements of 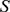 are independent given 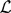. Similarly we define 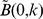 for non-linked 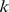:



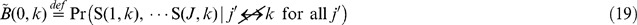
(19)

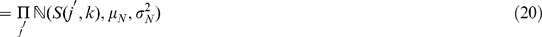
(20)


Lastly, we set the emission probabilities by normalizing the 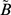
’s.
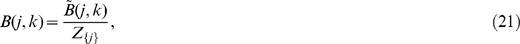
(21)

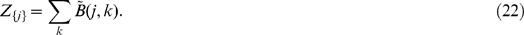
(22)


We define the HMM score for abstract sentence 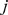 and figure 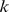, 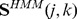, as the posterior probability that the state occupied on step 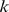, 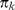, is state 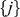.
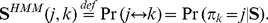
where the probability is with respect to the article’s derived HMM for the standard observation sequence (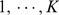). We use the *posterior decoding* procedure [31] to compute the HMM scores.

### Conditional Random Fields

Our HMM approach captures some properties of linkage features well, but fails to capture some others. Consider, for instance, the EdgesCrossed linkage feature and the transition 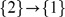. Whenever this transition is taken at step 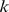, HMM semantics assert both 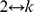 and 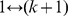, which induces a crossed edge in the linkage graph. Now, the HMM may learn a relatively small transition probability for 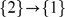, but only if other transitions from 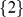 are more frequent in the training set. Standard HMM representations, however, provide no mechanism for generalization to transitions from other states using, for example, a common penalty for transitions that induce crossed edges. Such a general penalty is especially beneficial when learning transition probabilities, such as for 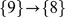, from less frequently visited states. Indeed, in our data set there are 98 total transitions from state 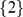 but only 19 from state 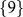. Conditional random fields [3] (CRFs), on the other hand, provide a mechanism for generalization through weights associated with a set of shared transition features.

Our CRF approach is similar in many respects to our HMM approach. Our CRFs also have SIS and FIS variants (we describe here the SIS variant), also associate linkage predictions with state sequence paths, and also generate a length 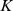 observation sequence 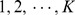 for an article with 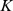 figures. Furthermore, like HMMs, the likelihood of a path through the model is proportional to the product of 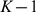 transition terms and 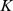 emission terms. There are, however, two key differences between our CRF and HMM methods. First, while each HMM state is associated with one or zero sentences, in CRFs we also have states associated with multiple sentences. These multi-sentence states enable us to link a figure with multiple sentences on a single path. Second, CRFs use a different representation transition affinity. While for HMMs the affinity of a transition is its transition probability, CRF transition affinity is given by a weighted sum of feature values. Sharing of features and weights enables information transfer across transitions.

A CRF for an article with 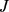 sentences has a state 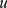 for every subset of the sentences 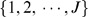 with size 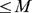. In our experiments we set 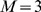. (Figure 7(b) shows an example CRF with 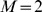.) We use 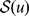 to refer to the set of sentences associated with state 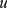 so, 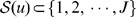 and 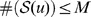 where 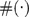 denotes set cardinality. The state sequence path for an article with 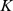 figures, 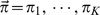, asserts that figure 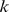 is linked with all abstract sentences in 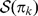. Thus, the number of abstract sentences linked with figure 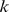 (the degree of 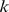), is 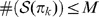, and the number of figures linked with abstract sentence 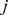 is 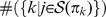 is 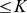. Since in the SIS construction, the degree of figure 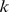 is entirely determined from 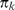 we are able to readily encode figure degree properties in CRF transition features. On the other hand, as the degree of sentence 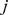 depends on the whole path, sentence degree properties are not as amenable to representation as transition features. One of the likely reasons that SIS representations outperform FIS representations is that the linkage feature FigureDegree is substantially more predictive of linkage than SentenceDegree (Table 2).

Our CRFs are parameterized by the transition feature weight vector 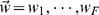, where 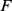 is the number of transition features, and weight 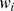 is associated with transition feature 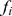. The weight vectors are set during training. Given 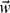, the CRF probability of the path 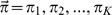 is proportional to the product of the start-state affinity (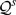), 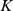 emission affinities (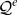), and 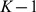 transition affinities (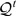):
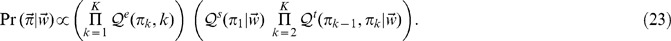
(23)


Here, 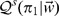 is the affinity for starting in state 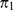, 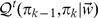 is the affinity for the transition 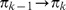, and the emission affinity 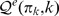 gives the affinity for linking figure 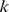 with sentences 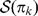. The emission affinities represent the textual coherence of the implied linkages, and are defined similarly to the emission probabilities of our HMMs. Also, the emission affinities do not depend on 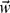, and so, as with HMM emission probabilities, they are not adjusted during CRF training.

#### Transition affinities

We now describe the transition features 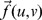 we use to represent the start-state and transition affinities. We represent the transition affinity for the transition from state 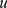 to 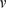 using the standard log-linear model:
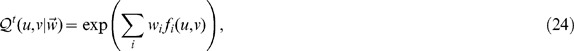
(24)where 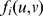 is the value of feature 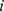 for 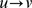. We have a similar representation for the start-state affinity:
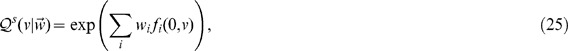
(25)where 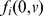 denotes the value of feature 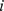 associated with starting in 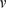. To simplify description of our features below, we define 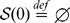.

We now describe our eight transition features. These features are closely related to the linkage features described above. However, as each transition only provides information on linkage of two neighboring figures, each feature 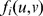 can only depend on two adjacent columns of the linkage matrix 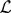 (see Figure 6). We have a group of four binary features related to the number of sentences in the destination state 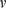. The names of these features all begin with “ FigureDegree” because the degree of figure 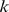
’s vertex in the graph representation is equal to 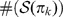. Each of these features is a binary test on 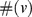, and for every state exactly one of these features is 1 and all other features are 0.
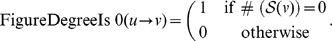


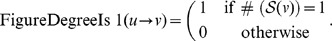


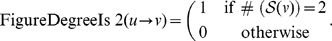


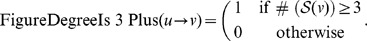



Our next feature, 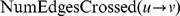, counts the number of crossed edges in the graph representation induced by the linkages implied by the transition. While this feature estimates the linkage feature 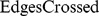 it will not count crossed edges that require more information about 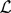 than what is implicit in the transition.
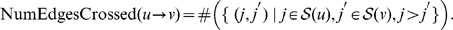



The last group of transition features (PreviousFigure, PreviousSentence and PrevSentAndFig) count the number of occurrences of neighborhood linkage patterns. Recall that 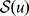 denotes the set of sentences associated with state 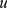. Thus, a path that includes transition 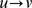 asserts that some figure 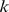 is linked with all the sentences 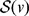 associated with the destination state 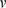, and that the previous figure 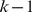 is linked with all the sentences 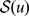 associated with the source state 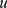. 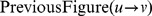 is the count of the number of times a sentence links with both figure 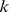 and the previous figure 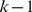:
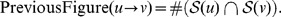



Similarly, 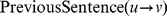 is the count of the number of times figure 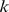 links with both sentence 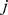 and the previous sentence 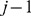:
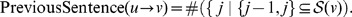



Note that 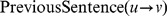 depends only on the sentences 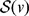 in the destination state. Although additional “previous sentence” counts can be inferred from linkages between figure 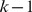 and source state sentences 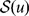, to prevent double counting we do not count them on 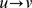 because they get counted on the transition into 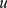. We define the last neighborhood feature, 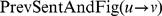, to be the count of the number of times figure 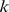 links with sentence 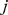 while the previous figure 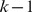 links with the previous sentence 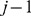:
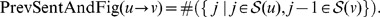



Unlike 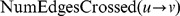, the values of the three neighborhood features are exact.

As an example, consider the transition 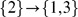 with 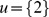 and 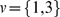. We have.
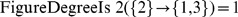




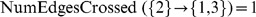
 (from 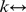
1 and 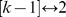),
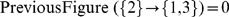


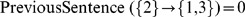



and 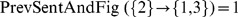 (from 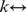
3 and 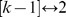).

#### Emission affinities

The emission affinity for symbol 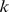 (for Figure 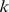) in state 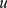, 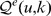, is based on the text coherence for all implied linkages and non-linkages. Emission of the symbol 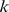 from state 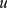 implies that sentences associated with 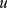, 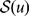, are linked with figure 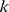 and all other sentences are not linked with 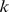. The matrix of CRF emission affinities 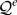 is closely related to the 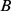 matrix of HMM emission probabilities:


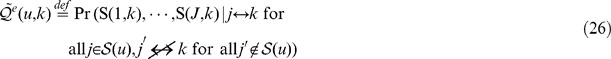
(26)



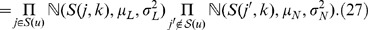
(27)where 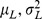, 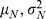, are as defined in the HMM section. We set 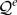 from 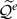 by normalizing:


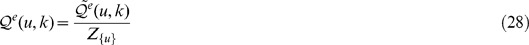
(28)



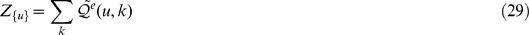
(29)


#### Prediction and learning

We use standard algorithms for learning weights and predicting linkages [3]. For learning we use gradient ascent to maximize the probability of training set state sequences. For prediction, we compute posterior distributions over 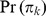 using the forward and backward dynamic programming passes.

### Performance Measures

#### Recall-precision, F1, and ROC curves

We compute precision (P), recall (R) and false-positive rate (FPR) for the linkage predictions of a set of sentence/figure instances:
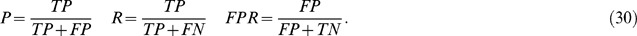
(30)


TP, TN, FP, FN are the number of true positive, true negative, false positive, and false negative predictions, respectively, where a “positive” instance is linked and a “negative” instance is not linked. A recall-precision curve plots R vs P, while a receiver operating characteristic (ROC) curve plots FPR vs R. Points on these curves are calculated by varying the threshold on linked score that separates positive predictions from negative predictions. The area under an ROC curve (AROC) ranges from 0.0 to 1.0 where the AROC of a random guess classifier is equal to 0.5. The F1 score of a classifier is the geometric mean of R and P: F1 = 2RP/(R+P).
